# WO_3_/α-Fe_2_O_3_/Bi_2_S_3_ ternary photoanode for improved oxygen evolution reaction in photoelectrochemical water splitting

**DOI:** 10.55730/1300-0527.3720

**Published:** 2025-02-17

**Authors:** Fatih TEZCAN, Abrar AHMAD, Gülfeza KARDAŞ

**Affiliations:** 1 Department of Chemistry and Chemical Process Technology, Vocational School of Technical Sciences at Mersin Tarsus Organized Industrial Zone, Tarsus University, Mersin, Turkiye; 2 Department of Chemistry, Faculty of Arts and Sciences, Çukurova University, Adana, Turkiye; 3 Department of Chemistry, Quaid-i-Azam University, Islamabad, Pakistan

**Keywords:** Photoelectrode, ternary heterojunction, photoelectrochemical water splitting, hydrothermal, successive ionic layer adsorption and reaction (SILAR) method

## Abstract

This study presents a ternary WO_3_/α-Fe_2_O_3_/Bi_2_S_3_ photoanode system suitable for photoelectrochemical water-splitting applications. WO_3_/α-Fe_2_O_3_ heterojunction is obtained using a hydrothermal approach, while Bi_2_S_3_ is deposited onto WO_3_/α-Fe_2_O_3_ via the successive ionic layer adsorption and reaction (SILAR) method. The cycle count is adjusted to determine the optimal photocatalytic photoanode. X-ray diffraction analysis confirms different morphologies and phases for the photoelectrodes: WO_3_ is deposited as plates with monoclinic phases, α-Fe_2_O_3_ as nanorods with hexagonal phases, and Bi_2_S_3_ in the form of nanoparticles (NPs) with orthorhombic phases. Solar light absorption spectra indicate that ternary WO_3_/α-Fe_2_O_3_/Bi_2_S_3_ photoanodes absorb a larger portion of the solar spectrum and display a large red shift in wavelength compared to binary WO_3_/α-Fe_2_O_3_ photoanodes. Chronoamperometric and electrochemical impedance spectroscopy measurements indicate that the as-prepared WO_3_/α-Fe_2_O_3_/Bi_2_S_3_ photoanode exhibits notable stability and low charge transfer resistance (R_ct_) compared to binary electrodes and pristine WO_3_ plates in faradaic photoelectrochemical conversion for the oxygen evolution reaction and S^−2^/S^2^ processes. Linear sweep voltammetry studies show that the WO_3_/α-Fe_2_O_3_/Bi_2_S_3_ photoanode, sensitized with 8 SILAR cycles, achieves the maximum photocurrent density of 5.777 mA.cm^−2^ at 1.0 V vs. RHE under 100 mW cm^−2^ simulated solar irradiation.

## 1. Introduction

The International Energy Agency forecasts a global primary energy consumption of 18.3 tonnes by 2035, with a growth rate of 1.37%. To meet this demand, at least 10 TW of zero-CO_2_ power-generating systems must be developed by 2050, posing a major challenge for 21^st^-century researchers [1]. Solar power is a promising renewable energy source that offers long-term solutions to energy and environmental issues. However, effective utilization requires addressing challenges related to energy collection, conversion, and storage. Hydrogen is considered the ideal future energy source due to its high conversion efficiency, low carbon emissions, and ease of transportation [2].

One of the most promising technologies for clean and sustainable hydrogen production is photoelectrochemical water splitting, which harnesses the 120,000 TW of solar radiation reaching the Earth’s surface [3]. Since the initial investigation of titanium dioxide (TiO_2_) nanocrystal as a photoanode for photoelectrochemical cell (PEC) into water splitting by Fujishima and Honda in 1972, numerous nanostructured photoanodes of α-Fe_2_O_3_[4], ZnO[5], WO_3_[6], and BiVO_4_[7] have been extensively researched for a similar purpose [8]. Among a number metal oxide-based semiconductors, WO_3_ stands out as a viable choice for PEC water splitting as it can absorb about 12% of the solar spectrum (band gap: 2.50–2.99 eV), low cost, moderate diffusion length (approximately 150 nm), good photostability, and high electron mobility (up to 12 cm^2^V^−1^S^−1^) [9,10]. However, the practical performance of the WO_3_ photoanode is still far from what is theoretically expected due to the short lifetime of excited states, improper conduction band-edge position, poor oxygen evolution reaction (OER) kinetics, and inefficient charge transport at the photoelectrode/electrolyte interface. Furthermore, α-Fe_2_O_3_ is similar to WO_3_ as a photoanode material for oxidation of the water in terms of its abundance, nontoxicity, sensitivity to the visible region of the sunlight’s spectrum, and positive conduction band position for water oxidation [11–13]. Nonetheless, the PEC performance of αFe^2^O^3^ is restricted by intrinsic drawbacks, including inefficient reaction kinetics for the oxygen evolution reaction, a high rate of carrier recombination, a relatively short hole diffusion length of 2/4 nm, and a positively located valence band with respect to the water reduction potential [14–16]. Generally, photocurrent density is defined by the capacity of the photoanode to yield the incident light, its charge separation-transport efficiency, and its PEC stability [9]. However, a single semiconductor oxide cannot completely fulfil all these requirements; hence, its PEC performance is limited. Consequently, multiple approaches have been explored to improve photoanode performance, including doping, cocatalyst deposition, the fabrication of large surface areas nanostructures, bandgap lowering, heterojunction architecture, and construction of composites [17–20]. Among these strategies, heterojunction formation is particularly promising due to its potential to improve PEC performance by combining two or more semiconductor materials with complementary photoactive properties [20,21].

Mao et al. synthesized WO^3^/α-Fe^2^O^3^ heterojunction photoanodes on a photoelectrode substrate using a surfactant-assisted sol-gel process and the electrodeposition method. The result showed that the coupling of WO_3_ with α-Fe_2_O_3_ results in a significant increase in visible light absorption from 480 to 600 nm, and the highest current density of 0.91 mA cm^−2^ was observed in sodium sulfate electrolyte, which is nine times greater than bare WO_3_ [22]. Sivula et al. successfully synthesized WO_3_/α-Fe_2_O_3_ heterojunctions by using the atmospheric pressure chemical vapor deposition method. WO_3_/α-Fe_2_O_3_ photoelectrode shows a 20% boost in the photoresponse as compared to bare WO_3_ [23]. Li et al. successfully developed WO_3_/α-Fe_2_O_3_ photoelectrode on an FTO substrate and a significant photocurrent density of 1.66 mA cm^−2^ was observed, which is six times higher than pristine WO_3_. The enhancement in photocurrent density was ascribed to increased light gathering efficiency alongside effective charge separation [24]. Nonetheless, these binary systems are impeded by restricted visible light absorption and reduced charge separation efficiency [25]. Recently, ternary heterojunctions have received significant attention due to their ability to broaden solar light absorption. Bai et al. successfully produced a novel ternary photoanode consisting of Bi nanoparticles (NPs) embedded in a WO_3_/ZnWO_4_ type II heterojunction using basic and inexpensive drop-casting and chemical impregnation procedures. PEC studies revealed that WO_3_/ZnWO_4_/Bi NPs exhibited a photocurrent density six times higher than that of bare WO_3_. The improved PEC performance was attributed to the creation of type II heterojunctions and the alteration of Bi NPs. The broad range not only boosted the absorption spectrum (WO_3_/ZnWO_4_) for visible light and improved carrier separation efficiency but also enhanced light capture ability through the local surface plasmon resonance action of Bi nanoparticles and the generation of hot electrons. [26]. Subrahmanyam et al. successfully synthesized a TiO_2_/CdS/BiSbS_3_ p-n heterojunction by combining the doctor blade method, SILAR, and the chemical bath deposition method (CBD). The ternary TiO_2_/CdS/BiSbS_3_ heterojunction exhibited the highest PEC performance as compared to binary TiO_2_/CdS and TiO_2_/BiSbS_3_ photoelectrodes. The improved PEC performance was attributed to the visible light absorption capability of ternary TiO_2_/CdS/BiSbS_3_ photoelectrode associated with TiO_2_, CdS, TiO_2_/CdS, and TiO_2_/BiSbS_3_ photoelectrode. Moreover, the favorable band edge positions of TiO_2_, CdS, and BiSbS_3_ play a vital role in effective charge separation and transfer [27]. Masoumi et al. designed a Fe_2_O_3_/WS_2_/WO_x_ photoanode for photoelectrochemical water splitting for light harvesting in a broad spectrum. Under back-side illumination, the Fe_2_O_3_/WS_2_/WO_x_ photoanode achieved a photocurrent density of 2.1 mA cm^−2^ in 1.0 M NaOH electrolyte [28].

Our previous studies focused on binary heterostructures, such as α-Fe_2_O_3/_Bi_2_S_3_ and rosette-rod TiO_2_/Bi_2_S_3_ photoelectrodes, for photoelectrochemical hydrogen production [29,30]. These photoelectrodes exhibited photocurrent densities of 2.5550 mA cm^−2^ and 3.980 mA cm^−2^ vs. RHE under 100 mW cm^−2^. Although these binary electrodes demonstrated excellent photocatalytic performance, they utilized only a limited portion of the solar spectrum. Ternary heterostructures offer improved utilization of the solar spectrum, encompassing ultraviolet, visible, and near-infrared light. For instance, Liu et al. studied Bi_2_S_3_/BiVO_4_/TiO_2_ ternary photoelectrode for photodegradation organic dyes. Their findings suggested that incorporating BiVO_4_/TiO_2_ photocatalyst after Bi_2_S_3_ not only seriously enhanced the solar light absorption but also broadened the absorption spectrum [31]. Chen et al. developed a Bi_2_S_3_/MoS_2_/Bi_2_MoO_6_ ternary heterostructure for the catalytic performance of photoelectrocatalytic degradation. It demonstrated robust visible light harvesting due to the broadened absorption spectrum of the heterostructure [32].

In this study, the WO_3_/α-Fe_2_O_3_ heterojunction was successfully synthesized using a straightforward two-step hydrothermal method, followed by the deposition of Bi_2_S_3_ nanoparticles on the heterojunction surface via the SILAR technique to enhance charge separation and light absorption efficiency. Various SILAR cycles were performed to examine the light-harvesting efficiency and photoelectrochemical performance of the WO_3_/α-Fe_2_O_3_/Bi_2_S_3_ ternary photoelectrode, ultimately achieving the best-performing photoelectrode. Photoelectrochemical performance was evaluated under solar illumination by measuring current density, electrochemical double-layer resistance, and flat band potential (V_fb_) under solar illumination using linear sweep voltammetry (LSV), electrochemical impedance spectroscopy (EIS), and Mott–Schottky experiments. The most catalytic photoelectrode showed the highest photocurrent density of 5.777 mA cm^−2^ (1.0 V vs. RHE) under simulated solar irradiation.

## 2. Experimental procedure

### 2.1. Materials and chemicals

Sodium tungstate dihydrate (Na_2_WO_4_.2H_2_O, ≥99.0%), diammonium oxalate monohydrate (NH_4_)_2_ C_2_O_4_. 2H_2_O ≥99.0%), bismuth (III) nitrate pentahydrate (Bi(NO_3_)_3_·5H_2_O, 98%), ethylene glycol (C_2_H_6_O_2_ ≥ 99.0%), iron(III) chloride anhydrous (FeCl_3_, min 98%), urea (CO(NH_2_)_2_ 99.0–101.5%), and sodium sulfide nonahydrate (Na_2_S·9H_2_O) were all obtained from Sigma-Aldrich (Darmstadt, Germany). Sodium sulfite (Na_2_SO_3_ 95%), hydrochloric acid (HCl 37%), acetone (C_3_H_6_O 99.5%), and ethyl alcohol (C_2_H_5_OH 99.9%) were purchased from Merck (Rahway, NJ, USA). Fluorine-doped tin oxide (FTO, ~10 Ω/Sq) substrates were procured from Teknoma Company (İzmir, Türkiye). All chemicals used were of analytical grade and used as received, without purification. FTO substrates were cleaned with distilled water (diluted with detergent) followed by ethanol, acetone, and distilled water, each for 10 minutes in an ultrasonic bath.

### 2.2. Synthesis of WO_3_ plates

The synthesis of WO_3_ plates on the FTO was directly carried out using a hydrothermal approach. First, 0.247 g of sodium tungstate dihydrate was dissolved in 30 mL of the distilled water, followed by a dropwise addition of 3 M HCl (10 mL), with continuous stirring for 15–20 min. The as-prepared solution was then mixed with 30 mL diluted solution of diammonium oxalate monohydrate (55 mM) while stirring vigorously for 30 min. The solution was then transferred to an autoclave, positioning the conductive face of the FTO upward. The hydrothermal reaction was carried out at 140 °C for 5 h. After reaching room temperature, the WO_3_ plates seeded on the FTO substrates were removed, completely washed with distilled water and ethanol, and subsequently dried at 60 °C. Finally, the prepared WO_3_ plates were annealed for 5 h at 450 °C, with a heating program of 10°C/min to 450 °C under ambient conditions.

### 2.3. Synthesis of WO_3_ /α-Fe_2_O_3_ photoelectrode

α-Fe_2_O_3_ was synthesized on WO3 plates using a hydrothermal method. In 50 mL of distilled water, 0.975 g of iron (III) chloride anhydrous and 0.54 g of urea were dissolved and mixed thoroughly until a clear solution was achieved. The electrolyte was added to the autoclave containing the WO_3_ plates grown on the FTO. The hydrothermal reaction was conducted at 100 °C for 12 h, resulting in the formation of *β*-FeOOH. The photoelectrodes were then annealed at 550 °C for 5 h, with a heating program of 10°C/min to 550 °C, to attain α-Fe_2_O_3_ grown over the WO_3_ plates.

### 2.4. Synthesis of WO_3_ /α-Fe_2_O_3_/Bi_2_S_3_ photoelectrode

The deposition of Bi_2_S_3_ over a WO_3_/α-Fe_2_O_3_ photoelectrode was carried out using the SILAR method. Initially, the as-prepared WO_3_/α-Fe_2_O_3_ photoelectrode was dipped in a 0.05 M ethylene glycol solution of bismuth (III) nitrate pentahydrate for 1 min to deposit Bi^3+^ ions. The electrode was then cleaned with distilled water to remove excess Bi^2+^ ions. The sample was immersed in 0.1 M Na_2_S for 1 min to chemically bond S^−2^ ions, followed by a reaction with previously deposited Bi^3+^ ions to enable the deposition of Bi_2_S_3._ One SILAR cycle was completed by washing the sample again with distilled water to remove weakly attached Bi_2_S_3_. Different SILAR cycles (4, 6, 8, and 10 cycles) were performed to obtain several WO_3_/α-Fe_2_O_3_/Bi_2_S_3_ photoelectrodes with varying Bi_2_S_3_ deposition cycles.

### 2.5. Photoelectrode characterizations

The crystalline phase composition of photoelectrodes was analyzed on X-ray diffractometers (PANalytical Empyrean, XRD) (Kraków, Poland). A field emission scanning electron microscope (FEI Quanta 650 Field Emission SEM) (Oregon, USA) and an electron probe energy dispersive X-ray spectrometer (EDS) were used to analyze the surface morphology and elemental composition of the photoelectrodes. UV-visible spectra were attained using a UV-vis spectrophotometer (Agilent, Cary 7000) (Santa Clara, USA) to evaluate the optical absorption capabilities within the wavelength spectrum of 350–1000 nm.

#### 2.5.1. Photoelectrochemical measurements

PEC measurements were conducted with a conventional three-electrode setup using a CHI 660D electrochemical analyzer. The as-prepared photoelectrodes, a saturated Ag/AgCl (3 M KCl), and a Pt sheet (1 × 1cm) were used as a working electrode (0.80 × 1.25 cm), reference electrode, and counter electrode, respectively. 0.1 M Na_2_SO_3_ and 0.1 M Na_2_S were chosen as electrolytes. Linear sweep voltammetry measurements were carried out in a potential window ranging from −0.8 to 0.4 V vs. Ag/AgCl at a scan rate of 5 mV s^−1^. A solar simulator (Sunlite TM Solar Simulators, M-SLSS, AM 1.5 filter, 300 W) was used for standard solar light irradiation, and the solar power intensity was adjusted to 100 mW cm^−2^. The open circuit potential (EOCP) of samples was measured at approximately −0.6 V. EIS measurements were conducted at 0.0 V versus Ag/AgCl with a 5 mV amplitude, frequencies ranging from 10^5^ to 10^−1^ Hz under 100 mW cm^−2^ illumination. The ZView software was utilized to fit the EIS data and extract photoelectrochemical parameters. A chronoamperometric measurement was performed at 0.0 V vs. Ag/AgCl overpotential for 300 s under 100 mW cm^−2^ solar light. Mott–Schottky measurements were conducted with an AC amplitude of 5 mV at 500 Hz in the dark after establishing electrochemical equilibrium at the electrochemical double layer for 30 min.

The photoelectrode photoconversion efficiency was calculated using the applied biased photon-to-current efficiency (ABPE) as shown in equation (1) [33]:


(1) $$\text{Photoconversion efficiency }(\%)=J_{ph}\frac{1.23-V}{p_{total}}\times 100,$$

where *J_ph_* is the measured current density, *V* is the applied overpotential vs. RHE, and *P_total_* is the applied solar power (100 mW/cm^2^).

To compare with previous studies, the photoelectrode potential measured versus the Ag/AgCl reference electrode was converted to the reversible hydrogen electrode (RHE) using the Nernst equation (2).


(2) $$E_{RHE}=E_{\text{Ag}/\text{AgCl}}+0.059pH+E_{\text{Ag}/\text{AgCl}}^{{}^{\circ}},$$

where *E_RHE_* is the potential for the total water reaction, *E_Ag/AgCl_* is the potential measured versus Ag/AgCl reference electrode, and *E_Ag/AgCl_* is the ordinary potential of Ag/AgCl reference electrode, which is equal to 0.197 V at 25 °C for 3.0 M KCl [34].

## 3. Results and discussions

### 3.1. Characterizations of the photoelectrodes

**Figure 1:**
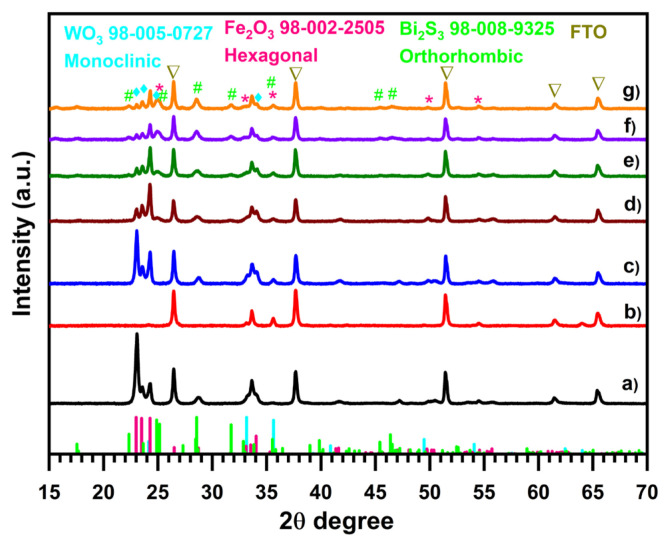
XRD patterns of WO_3_ (a) α-Fe_2_O_3_ (b), WO_3_/α-Fe_2_O_3_ (c) WO_3_/α-Fe_2_O_3_ /Bi_2_S_3_ 4 cyc (d), WO_3_/α-Fe_2_O_3_ /Bi_2_S_3_ 6 cyc (e), WO_3_/α-Fe_2_O_3_ /Bi_2_S_3_ 8 cyc (f), WO_3_/α-Fe_2_O_3_ /Bi_2_S_3_ 10 cyc (g).

Figure 1 illustrates the analysis of the crystal structure and phase purity of the prepared WO3, WO_3_/α-Fe_2_O_3_, and different WO_3_/α-Fe_2_O_3_/Bi_2_S_3_ heterojunctions using X-ray diffraction. The detected diffraction peaks indicate that the pristine WO_3_ electrode is in the monoclinic phase (reference code: ICSD:50727, *a=*7.297, *b=7.539, c=*10.515, space group P121/c1). The three main characteristic peaks at 23.11°, 23.58°, and 24.38° correspond to the (002), (020), and (200) diffraction planes of monoclinic WO_3_ [35–37]. The intensity and sharp diffraction patterns of the WO_3_ reveal outstanding crystallinity. Furthermore, the monoclinic structure appears in all the WO^3−^based samples with no additional peaks for any other phase of WO_3_. Upon the deposition of α-Fe_2_O_3_, new peaks appeared at 24.15°, 33.16°, 35.64°, 49.48°, and 54.08°, corresponding to (012), (104), (110), (024), and (116), respectively. The relative intensive 2θ° values of 33.16° and 35.64° relate to hexagonal hematite (α-Fe_2_O_3_) crystal structure (reference code: ICDD:00-033-0664, *a =* 5.034, *b = 5.034*, *c =* 13.748, space group R-3 c) [38–40]. Upon the deposition of the third Bi_2_S_3_ NPs layer on the WO_3_/α-Fe_2_O_3_ heterojunction by the SILAR method, additional new peaks appear at 2θ° values of 17.5, 23.76°, 25.06°, 25.26°, 28.70°, 31.89°, 33.01°, 35.69°, 45.64°, and 46.58°, corresponding to the (120), (011), (111), (103), (211), (212), (013), (402), (020) and (314) crystal plane of orthorhombic Bi_2_S_3_ (reference code: ICSD: 89325 *a =* 11.269, *b* = 3.972 *c =* 11.129, space group Pnma) [41,42]. However, the XRD pattern clearly shows that with the increase in the number of SILAR cycles, the peak for orthorhombic Bi2S3 becomes more pronounced.

**Figure 2:**
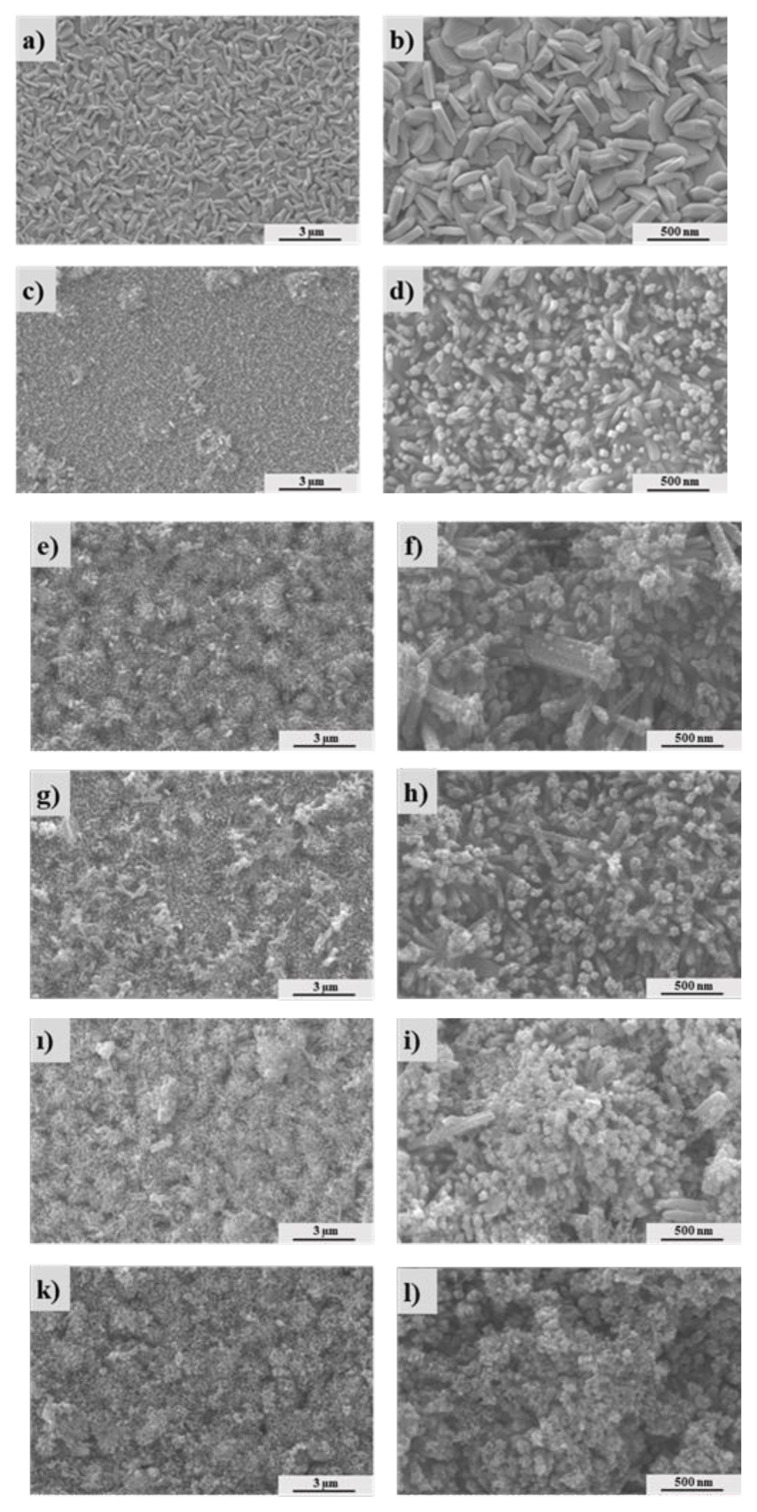
FESEM images of WO_3_ (a–b),WO_3_/α-Fe_2_O_3_ (c–d), WO_3_/α-Fe_2_O_3_/Bi_2_S_3_ 4cyc (e–f), WO_3_/α-Fe_2_O_3_ /Bi_2_S_3_ 6cyc (g–h), WO_3_/α-Fe_2_O_3_ /Bi_2_S_3_ 8cyc (1–i), WO_3_/α-Fe_2_O_3_ /Bi_2_S_3_ 10cyc (k–l).

FESEM images showing the morphology of WO_3_, WO_3_/α-Fe_2_O_3,_ and WO_3_/α-Fe_2_O_3_ /Bi_2_S_3_ are presented in Figure 2. Figures 2a and 2b show that the bare WO_3_ consists of plate arrays produced on the FTO substrate with a smooth surface, good density, and random vertical alignment. The dimensions of the WO_3_ plates are approximately 1823 × 578 × 291 nm. However, upon the hydrothermal deposition of α-Fe_2_O_3_, it can be seen in Figures 2c and 2d that tetragonal-shaped α-Fe_2_O_3_ nanorods (with radii of 40–60 nm) are uniformly grown over the entire WO_3_ surface. Figures 2e–2l show the FESEM images after the deposition of the third Bi_2_S_3_ layer. Bi_2_S_3_ NPs (15–18 nm) are homogeneously deposited over the entire α-Fe_2_O_3_ nanorods, not only on the tips but also along the sides of the α-Fe_2_O_3_. It can be inferred that as the number of SILAR cycles increases, the loading of Bi2S3 also increases, leading to the aggregation of Bi_2_S_3_ nanoparticles.

Table 1 shows the atomic percentage composition of elements in the photoelectrodes, as determined by EDS spectra. EDS results show a significant decrease in the atomic percentage of W as we move from bare WO_3_ towards the binary (WO_3_/α-Fe_2_O_3_) and ternary (WO_3_/α-Fe_2_O_3_/Bi_2_S_3_) heterojunctions. This implies that the α-Fe_2_O_3_ nanorods and Bi_2_S_3_ are regularly deposited on the WO_3_ plates, decreasing the detection limit of tungsten. As the number of SILAR cycles increases, the atomic percentages of Bi and S grow significantly, indicating that more Bi_2_S_3_ is being deposited.

**Figure 3:**
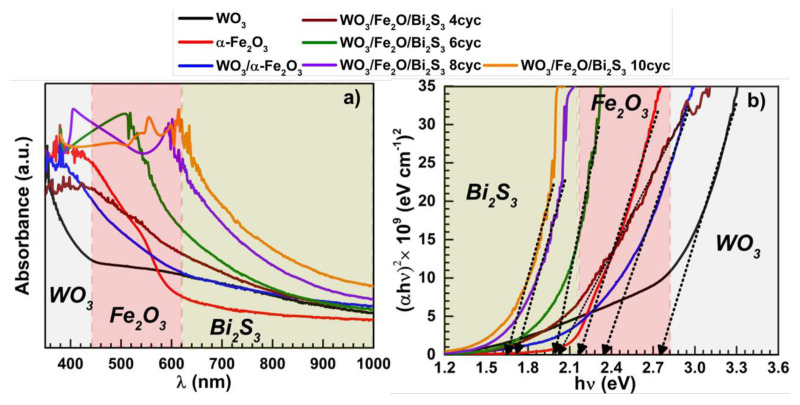
Uv-vis spectra (a) and Tauc plots (b) of the photoelectrodes.

To explore the influence of heterostructure on visible light harvesting efficiency, UV-vis optical absorption spectra of the as-prepared WO_3_, WO_3_/α-Fe_2_O_3_, and WO_3_/α-Fe_2_O_3_/Bi_2_S_3_ were recorded within an optical window ranging from 350 to 1000 nm, as shown in Figure 3a. The UV-vis spectra clearly show that the absorption edge for bare WO_3_ is at about 450 nm. As WO_3_ is modified with α-Fe_2_O_3_, it causes a red shift, pushing the absorption edge into the visible range of the solar spectrum. Adding Bi_2_S_3_ nanoparticles further shifts the absorption to about 920 nm and slightly boosts the absorption strength due to the small band gap of Bi_2_S_3_. The UV-visible data indicates that light absorption efficiency improves with more SILAR cycles due to the increased Bi_2_S_3_ deposition. The band gap (Eg) of a semiconductor represents the energy necessary for an electron to transition between the energy levels within the material. This value can be determined using a Tauc plot by plotting (*αhν*)2 versus *hν* (Figure 3b). The *E_g_* of pristine, binary, and ternary photoelectrodes is calculated using the following the Tauc equation (3):


(3) $${\left(\alpha hv\right)}^{1/n}=A(hv-E_{g}),$$

where α, *h*, v, *n*, and A are the coefficient of absorption, the Planck constant, the frequency of light, the direct band transition of semiconductor (*n = 1*), and the experimental constant, respectively. The E_g_ values was calculated as 2.76, 2.17, 2.23, 1.95, 1.88, 1.67, and 1.61 eV for WO_3_, α-Fe_2_O_3_, WO_3_/α-Fe_2_O_3_, WO_3_/α-Fe_2_O_3_/Bi_2_S_3_ 4cyc, WO_3_/α-Fe_2_O_3_/Bi_2_S_3_ 6cyc, WO_3_/α-Fe_2_O_3_/Bi_2_S_3_ 8cyc, and WO_3_/α-Fe_2_O_3_/Bi_2_S_3_ 10cyc samples, respectively.

### 3.2. Photoelectrochemical performance of photoelectrodes

**Figure 4:**
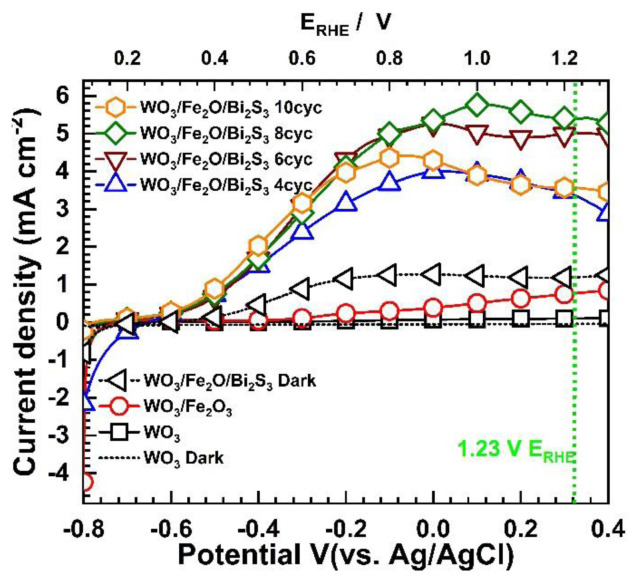
LSV curves of WO_3_, WO_3_/α-Fe_2_O_3_, and various SILAR cycles.

Figure 4 shows the LSV measurements of the photoelectrodes at a scan rate of 5 mV within the potential range of −0.8 to 0.4 V (vs. Ag/AgCl) under 100 mW cm^−2^ solar light simulation in a mixed aqueous solution containing 0.1 M Na_2_SO_3_ and 0.1 M Na_2_S.

LSV results show that bare WO_3_ plates and binary WO_3_/α-Fe_2_O_3_ heterojunctions show very low photocurrent densities of 0.099 mA cm^−2^ and 0.739 mA cm^−2^ at 1.23 V_RHE_ for photoelectrochemical water splitting. The photocurrent density in the binary WO_3_/α-Fe_2_O_3_ heterojunction is seven times higher than pristine WO_3_, but this increase is not sufficient for efficient photoelectrochemical water splitting. However, upon deposition of Bi_2_S_3_ through the SILAR cycles, the catalytic behavior was enhanced, even under dark conditions. Furthermore, under solar light irradiation, a significant improvement in the photocurrent density was observed, with a maximum photocurrent density of 5.416 mA cm^−2^ at 1.23 V_RHE_ for WO_3_/α-Fe_2_O_3_/Bi_2_S_3_ photoelectrode, followed by WO_3_/α-Fe_2_O_3_/Bi_2_S_3_ 6cyc (4.980 mA cm^−2^ at 1.23 V_RHE_), WO_3_/α-Fe_2_O_3_/Bi_2_S_3_ 10cyc (3.570 mA cm^−2^ at 1.23 V_RHE_) and WO_3_/α-Fe_2_O_3_/Bi_2_S_3_ 4cyc (3.460 mA cm^−2^ at 1.23 V_RHE_).

However, upon deposition of Bi_2_S_3_ through the SILAR cycle, a significant improvement in the photocurrent density was observed, with a maximum photocurrent density of 5.416 mA cm^−2^ at 1.23 V_RHE_ for WO_3_/α-Fe_2_O_3_/Bi_2_S_3_ photoelectrode, followed by WO_3_/α-Fe_2_O_3_/Bi_2_S_3_ 6cyc (4.980 mA cm^−2^ at 1.23 V_RHE_), WO_3_/α-Fe_2_O_3_/Bi_2_S_3_ 10cyc (3.570 mA cm^−2^ at 1.23 V_RHE_) and WO_3_/α-Fe_2_O_3_/Bi_2_S_3_ 4cyc (3.460 mA cm^−2^ at 1.23 V_RHE_). The enhancement in PEC performance is attributed to Bi_2_S_3_’s broad light absorption range and high absorption coefficient [37,43]. LSV results indicate that increasing the SILAR cycles from 4 to 8 boosts the photocurrent density. However, increasing beyond 8 to 10 cycles reduces the photocurrent density, indicating that the limit is reached at 8 cycles, and additional cycles do not impact PEC performance. The reduction in photocurrent density after 8 SILAR cycles of deposition is attributed to excessive loading of Bi_2_S_3_, which increases recombination sites and causes electron loss within the ternary heterostructure.

**Figure 5:**
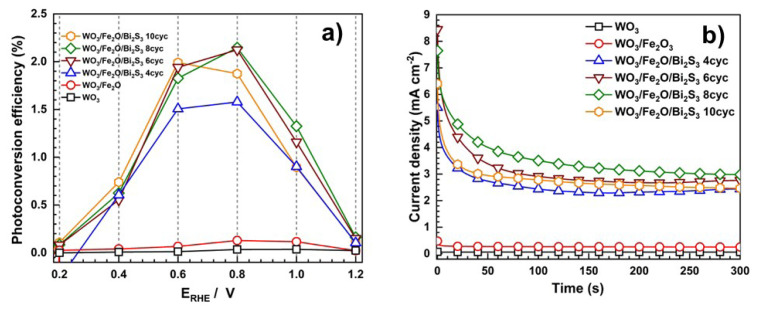
Photoconversion efficiency (a) and chronoamperometric measurements (b) of WO_3_, WO_3_/α-Fe_2_O_3_, and WO_3_/α-Fe_2_O_3_/Bi_2_S_3_ photoelectrodes with various SILAR cycle deposits under 100 mW cm^−2^ solar light illumination.

As shown in Figure 5, we obtained the photoconversion efficiency by applying biased photon-to-current conversion efficiency (ABPE). It shows that the applied potential and solar irradiation intensity of 100 mW cm^−2^ convert both the bias potential and photon energy to the current density of the photoelectrode. The photoconversion efficiencies of bare WO_3_ plates and binary WO_3_/α-Fe_2_O_3_ photoelectrodes are 0.034% at 0.8 V _RHE_ and 0.128% at 0.8 V _RHE_, respectively. This indicates that the formation of binary heterojunction by combining WO_3_ plates and α-Fe_2_O_3_ results in an improvement in the photoconversion efficiency, but it is not sufficient due to the limited solar light absorption capability of the binary system. Furthermore, among the ternary photoelectrodes, WO_3_/α-Fe_2_O_3_/Bi_2_S_3_ 8cyc exhibits the best photoconversion efficiency of 2.15% at 0.8 V_RHE_ followed by WO_3_/α-Fe_2_O_3_ /Bi_2_S_3_ 6cyc (2.12% at 0.8 V_RHE_), WO_3_/α-Fe_2_O_3_/Bi_2_S_3_ 10cyc (1.99% at 0.6 V_RHE_), and WO_3_/α-Fe_2_O_3_/Bi_2_S_3_4cyc (0.35% at 0.8 V_RHE_).

To evaluate the photoresponse and stability of the photoanodes, chronoamperometric measurements (j–t) were conducted at an applied voltage of 1.23 V vs. RHE under 100 mW cm^−2^ solar light illumination, as shown in Figure 5b. The results indicate that the pristine WO_3_ and binary WO_3_/α-Fe_2_O_3_ electrodes exhibit a nearly steady photocurrent throughout the measurement. However, ternary WO_3_/α-Fe_2_O_3_/Bi_2_S_3_ photoelectrodes initially exhibit a high photocurrent due to the sudden separation of electrons and holes upon illumination, followed by instant degeneration until a steady photocurrent is achieved. This transient decline is primarily attributed to electron–hole recombination [44]. Furthermore, Bi_2_S_3_ tends to experience photocorrosion (Bi_2_S_3_ + 3h^+^→ Bi^3+^ + S), which results in a decrease in photocurrent density [45]. Nevertheless, the ternary photoanodes demonstrate only a minimal decrease in photocurrent density, suggesting good stability under solar illumination. The chronoamperometry results also show that WO_3_/α-Fe_2_O_3_/Bi_2_S_3_ 8cyc performs the highest photocurrent, followed by WO_3_/α-Fe_2_O_3_/Bi_2_S_3_ 6cyc, WO_3_/α-Fe_2_O_3_/Bi_2_S_3_10cyc (1.99% at 0.6V_RHE_), and WO_3_/α-Fe_2_O_3_/Bi_2_S_3_ 4cyc—consistent with the LSV results.

**Figure 6:**
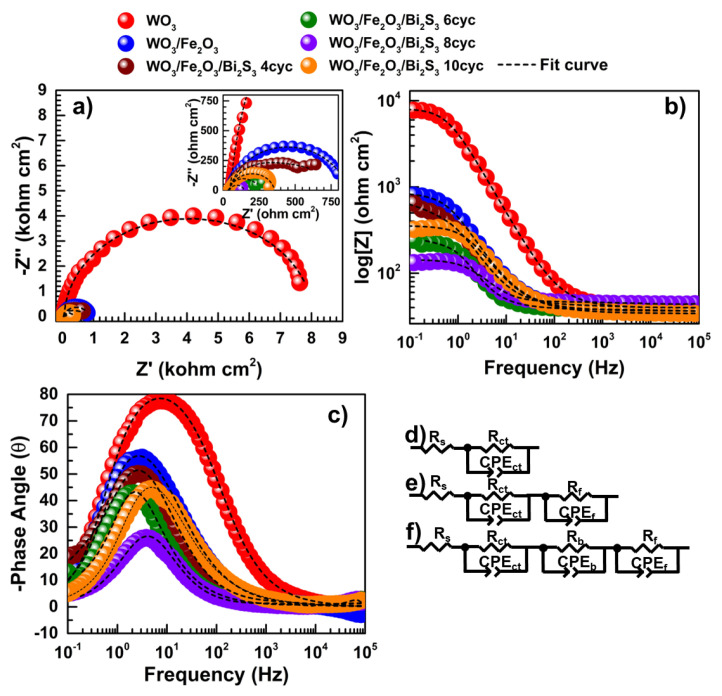
Nyquist (a), Bode (b), and phase angle-frequency (c) plots of WO_3_, WO_3_/α-Fe_2_O_3_, and WO_3_/α-Fe_2_O_3_/Bi_2_S_3_ photoelectrodes with various SILAR cycles under 100 mW cm^−2^ solar irradiation. The suggested electrical equivalent circuit of WO_3_ (d), WO_3_/Fe_2_O_3_ (e), and WO_3_/α-Fe_2_O_3_/Bi_2_S_3_ photoelectrodes various SILAR cycles (f).

EIS measurements were performed at 0.0 V versus Ag/AgCl under solar light irradiation of 100 mW cm^−2^. The goal was to identify the sources of resistance between the photoelectrode and electrolyte, observe electron/hole recombination, and analyze charge kinetics within the photoelectrodes. The Nyquist, Bode, and phase angle-frequency plots for the photoelectrodes are presented in Figures 6a–6c. A smaller semicircle radius typically results in lower photoresistance or charge transfer resistance at the electrode/electrolyte interface. The Nyquist curve depicts one, two, and three depressed semicircles for the bare WO_3_, binary (WO_3_/α-Fe_2_O_3_), and ternary photoelectrodes (WO_3_/α-Fe_2_O_3_/Bi_2_S_3_), respectively (Figure 6a). This suggests that binary and ternary samples cause a reduction in the supplies of resistance on the OER and S^−2^/S^2^ over the energy levels of photoelectrode/electrolyte assessed by the pristine WO_3_ plates. However, the binary and ternary photoelectrodes consist of two and three depressed semicircles, respectively. A smaller diameter of depressed semicircles increases electron transfer between energy levels of the semiconductor and lowers overall resistance.

Moreover, the Bode plot reveals the overall resistance based on the difference between the initial and final values, as shown in Figure 6b. The initial value of the Bode plot matches uncompensated resistances, implying electrolyte resistance. As seen in Figure 6b, these initial values are similar among the photoelectrodes. Therefore, the last point of the y-axis demonstrates the overall resistance of photoelectrodes. The lower |Z| mode of the samples suggests increasing photoelectrochemical performance on the OER and S^−2^/S^2^ at the double layer. Both binary and ternary photoelectrodes exhibit reduced resistance compared to pristine WO_3_ plates. Moreover, the ternary WO_3_/α-Fe_2_O_3_/Bi_2_S 8cyc electrode shows the lowest overall resistance, followed by WO_3_/α-Fe_2_O_3_/Bi_2_S 6cyc, WO_3_/α-Fe_2_O_3_/Bi_2_S 10cyc and WO_3_/α-Fe_2_O_3_/Bi_2_S 4cyc. Likewise, the magnitude of the phase angle indicates increasing resistance supplies of semiconductors to photoelectrochemical performance. Bi_2_S_3_ deposition causes a shifting of the maximum phase angle value in the lower frequency region. Charge transfer resistance (R_ct_) is associated with electron transfer among the semiconductor/electrolyte boundaries on the OER and S^−2^/S_2_ by applying photoelectrochemical energy. This suggests that Bi_2_S_3_ deposition over the binary electrode reduces R_ct_ among the ternary electrodes. The WO_3_/α-Fe_2_O_3_/Bi_2_S_3_ 8cyc electrode achieves the lowest R_ct_ by shifting the top of the phase angle to a lower frequency region. It can be concluded that the electronic transition causes a similar spin among the energy levels of Bi_2_S_3_ at a higher frequency, which in turn decelerates the recombination process and prolongs the electron’s resting time at the low-frequency region of Bi_2_S_3_.

The EIS results are fitted to a suggested electrical equivalent circuit (Figures 6d–6f) to calculate photoelectrochemical parameters, such as resistances and constant phase elements (CPE), as presented in Table 2. The analysis indicates that the electrical equivalent circuits of pristine WO_3_ plates, binary, and ternary photoelectrodes correspond to one, two, and three time constants, respectively. The equivalent circuit components are as follows: R_s_ represents solution resistance (uncompensated resistance related to solution), R_ct_ corresponds to charge transfer resistance (at the higher frequency), R_b_ is barrier resistance (at the low frequency), and R_f_ corresponds to film resistance (at the low frequency). The polarization resistance (R_p_) indicates overall resistance depending on OER and S^−2^/S^2^ at the double layer and it is the sum of all resistances (R_p_ = R_ct_ + R_b_ + R_f_). R_ct_ is associated with faradaic photoelectrochemical conversion on the electrode boundary. According to the calculated R_ct_ results, the ternary WO_3_/α-Fe_2_O_3_/Bi_2_S 8cyc performs the lowest R_ct_ compared to binary electrodes and pristine WO_3_ plates, indicating its superior performance for OER and S^−2^/S^2^ processes. Additionally, the C_CPEct_ value of WO_3_/α-Fe_2_O_3_/Bi_2_S_3_ 8cyc suggests that further charged transporters and compounds to oxidize accumulate at the electrode/electrolyte interface. The R_b_ parameter, available for ternary electrodes, shows that WO_3_/α-Fe_2_O_3_/Bi_2_S_3_ 8cyc exhibits the lowest R_b_ resistance, enhancing charge transport across the three-layered structure. Furthermore, R_f_ values tend to decrease with an increasing number of Bi_2_S_3_ SILAR cycles, attributed to enhanced electron transport enabled by Bi_2_S_3_’s broad solar light absorption. Overall, the WO_3_/α-Fe_2_O_3_/Bi_2_S_3_ 8cyc electrode achieves the lowest total resistance (R_p_) for OER and S^−2^/S^2^ at the double layer under photoelectrochemical energy input.

**Figure 7:**
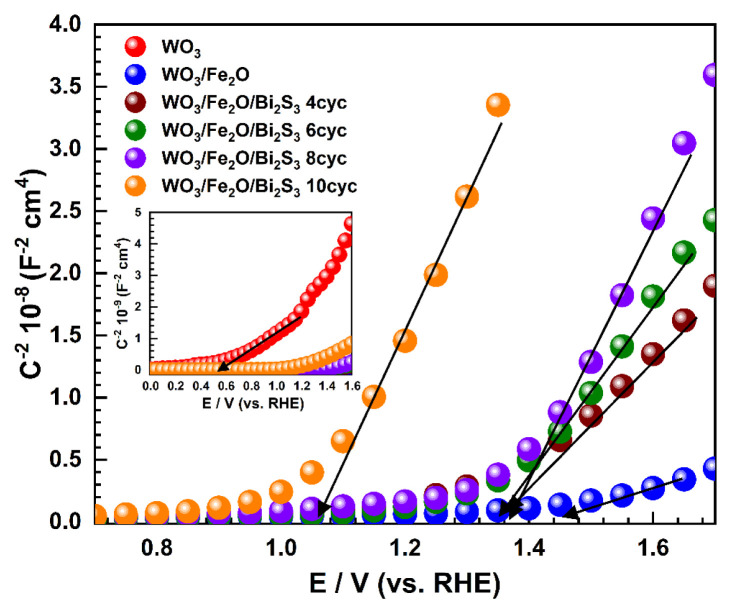
Mott–Schottky curves of WO_3_, WO_3_/α-Fe_2_O_3_, and WO_3_/α-Fe_2_O_3_/Bi_2_S_3_ photoelectrodes with various SILAR cycles under dark conditions.

The photoelectrochemical OER and S^−2^/S^2^ processes occur at electrode/electrolyte boundaries, and their properties—such as charge carrier density (*N_D_*), depletion layer (*W*), and flat band potential (*V_fb_*)—can be obtained using Mott–Schottky measurements. Figure 7 demonstrates the Mott–Schottky measurement of the photoelectrodes after a half-hour of steady state under dark conditions. According to Mott-Schottky theory, positive and negative slopes of the curve indicate n-type and p-type semiconductor materials, respectively. The results confirm that all photoelectrodes exhibit n-type semiconductor behavior, enabling the use of photoanodes in OER and S^−2^/S^2^ processes.

A *V_fb_* parameter of photoelectrode corresponds to the conductor band of the semiconductor, and it can be calculated using the following equation (4):


(4) $$\frac{1}{C^{2}}=\frac{2}{qɛ\;{ɛ}_{o}N_{D}}\left[V-V_{fb}-\frac{K_{B}T}{q}\right].$$

Here *q* represents the electron charge (1.6 × 10^−19^ C), *C* is the capacitance of the semiconductor, ɛ is the dielectric constant of the material, ɛ_o_ is the permittivity in a vacuum (8.85 × 10^−14^ F/cm), *k_B_* is the Boltzmann constant (1.38 × 10^−23^J.K^−1^), *V* is the applied bias potential, and *T* is the absolute temperature. *V_fb_* is calculated by extrapolating the curves to the x-axis. *N_d_* indicates the electronic properties of photoelectrode immersed in an electrolyte. It can be calculated from the slope of the curve (S) using the following the equation (5):


(5) $$N_{D}=\frac{2}{qɛ\;{ɛ}_{o}\;\text{S}}.$$

According to Table 3, the V*_fb_* of WO_3_ plates is approximately 0.533 V. When α-Fe_2_O_3_ is deposited onto the WO_3_ plates, the V*_fb_* of the binary electrode shifts to a more positive value (1.390 V), indicating a shift in the Fermi energy level. This more positive V*_fb_* increases the chemical potential gradient at the photoelectrode/electrolyte interface, thereby enhancing charge separation and transport of photoinduced electron-hole pairs across the energy levels [46]. Therefore, this suggests that α-Fe_2_O_3_ nanorods enhance the photocatalytic performance at the binary electrode/electrolyte interface. However, the ternary electrodes exhibit a shift to a more negative Fermi level upon Bi_2_S_3_ deposition on the binary electrode. This indicates that the fermi level of Bi_2_S_3_ is more negative than that of α-Fe_2_O_3_ nanorods due to the increasing SILAR cycle deposition of Bi_2_S_3_, which leads to a negative shift in *V_fb_*. Generally, a higher *N_D_* at the photoelectrode/electrolyte interface correlates with a lower slope in the Mott-Schottky curve. α-Fe_2_O_3_ nanorods raise *N_D_* compared to pure WO_3_ plates at the binary electrode, which makes charge transfer better across the binary interface. In ternary electrodes, the *N_D_* value is enhanced with increasing SILAR cycles of Bi_2_S_3_ deposition. However, beyond a certain point, further SILAR cycles of Bi_2_S_3_ deposition cause a decrease in *N_D_* values in the ternary electrodes. Furthermore, the width of the depletion region (W) can be calculated using equation (6).


(6) $$W=\left[\frac{2ɛ\;{ɛ}_{o}}{qN_{D}}(V-V_{fb})\right].$$

The electrolyte influences the valence band’s energy level under light conditions, causing significant band bending at the double layer [47,48]. The magnitude of band bending supports the charge separation at the double layer, contributing to the enhanced PEC performance of photoelectrodes [49]. This suggests that an increased depletion width (W) correlates with improved PEC performance of the ternary electrodes.

### 3.2. Photoelectrochemical water splitting mechanism

**Figure 8:**
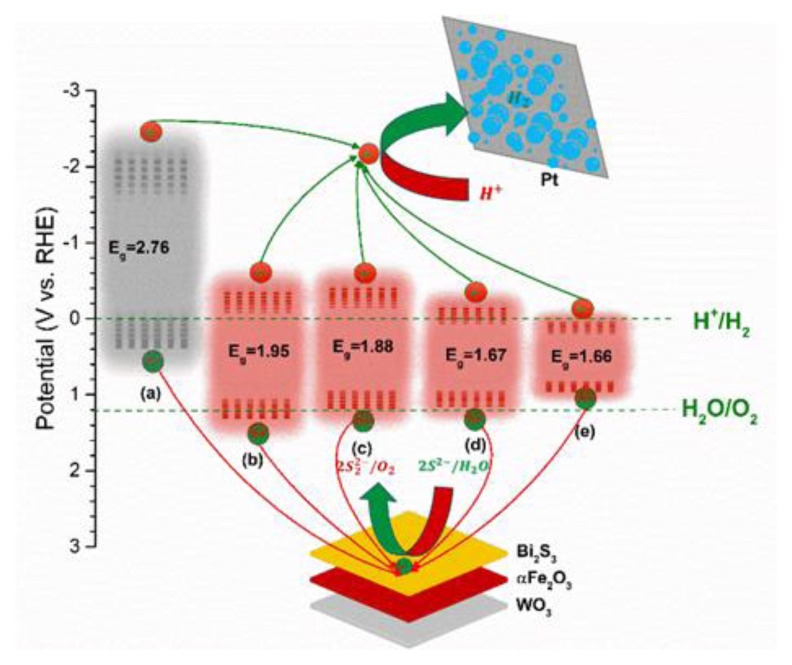
Energy level diagram of the photoelectrodes.

Figure 8 shows the photoelectrode valance band (VB) and conduction band (CB) energy levels of the photoelectrodes, illustrating the charge transfer mechanism in the photoelectrochemical water splitting process. These energy levels are determined using the E_g_ from the Tauc plot and the V_fb_ from the Mott–Schottky results. As seen in Figure 8, the VB value shifts to a negative energy level with binary and ternary photoelectrodes. Under solar irradiation, initially, electron transfer is performed from ternary photoelectrode VB to CB, leaving a positive hole over the VB. This positive hole suddenly attacks H_2_O molecules on the photoelectrode surface in the OER and S^−2^/S^2^ processes. Meanwhile, the excited electrons in the CB migrate to the cathode, where they reduce H^+^ ions to H_2_ molecules via the hydrogen evolution reaction (HER) on the Pt electrode. Therefore, O_2_ and H_2_ are produced at the photoanode and cathode surfaces, respectively. A necessary limit on input energy can be determined by the OER process with bias potential and solar light, and it depends on the E_g_ values of semiconductors. A lower E_g_ can reduce the required input energy and improve photocatalytic performance in PEC systems. Therefore, the ternary photoelectrodes benefit from having a lower E_g_. However, a low E_g_ value alone may not guarantee high PEC performance. This is also influenced by the alignment of the VB energy level with the H_2_O/O_2_ oxidation energy level. For efficient OER, the semiconductor’s VB must be more positive (i.e., at a higher energy level) than the H_2_O/O_2_ oxidation energy level. According to the energy level diagram, the VB levels of WO_3_ plates and WO_3_/Fe_2_O_3_/Bi_2_S_3_ 10cyc electrodes are more negative than the H_2_O/O_2_ oxidation energy level, suggesting they are unsuitable as photoanodes for photoelectrochemical water splitting. In contrast, the VB of WO_3_/α-Fe_2_O_3_/Bi_2_S_3_ 8cyc is closest to the H_2_O/O_2_ oxidation energy level, minimizing the required energy input and enhancing photocatalytic performance for photoelectrochemical hydrogen production.

## 4. Conclusions

In this study, ternary WO_3_/α-Fe_2_O_3_/Bi_2_S_3_ photoanodes on an FTO substrate were synthesized by combining the hydrothermal and SILAR approaches. The WO_3_/α-Fe_2_O_3_/Bi_2_S_3_ photoanode shows excellent light absorption response, covering a wide range of the solar spectrum with an absorption edge at about 920 nm. FESEM images reveal that WO_3_ plates are uniformly deposited on the FTO surface, followed by α-Fe_2_O_3_ deposition in tetragonal nanorods to shape the entire surface of WO_3_ plates, while Bi_2_S_3_ NPs are uniformly deposited over the α-Fe_2_O_3_ nanorods not only on the tips but also on the sides. PEC performance studies show that the ternary WO_3_/α-Fe_2_O_3_/Bi_2_S_3_ 8Cyc sample exhibits a maximum photocurrent density of 5.777 mA cm^−2^ (1.0 V vs. RHE), which is significantly higher than that of the binary (WO_3_/α-Fe_2_O_3_) and pristine WO_3_ photoelectrodes. Chronoamperometric and EIS measurements indicate that the as-prepared WO_3_/α-Fe_2_O_3_/Bi_2_S_3_ photoanode shows good stability with low charge transfer resistance (R_ct_) as compared to binary electrodes and pristine WO_3_ plates in faradaic photoelectrochemical conversion for OER and S^−2^/S^2^ processes. The findings reveal that this promising ternary semiconductor structure can significantly enhance photoelectrocatalytic efficiency and has high potential for use in solar cells and photocatalytic applications.

Table 4 represents similar ternary photoanode combinations with WO_3_/α-Fe_2_O_3_/Bi_2_S_3_. It shows that the WO_3_/α-Fe_2_O_3_/Bi_2_S_3_ photoanode exhibits enhanced photocatalytic activity in OER for photoelectrochemical water splitting.

## Figures and Tables

**Table 1:** EDX results of the samples.

	Atomic percentage (%)						
Sample	Bi	S	Fe	W	O	F	Sn
WO_3_	-	-	-	32.05	54.84	2.12	10.98
WO_3_/Fe_2_O_3_	-	-	20.89	29.99	65.33	-	10.11
WO_3_/Fe_2_O_3_/Bi_2_S_3_ 4cyc	3.67	8.16	20.00	6.22	55.87	-	10.00
WO_3_/Fe_2_O_3_/Bi_2_S_3_ 6cyc	4.74	10.84	20.06	5.87	50.83	-	10.01
WO_3_/Fe_2_O_3_/Bi_2_S_3_ 8cyc	6.89	13.78	21.85	2.10	44.52	-	10.8
WO_3_/Fe_2_O_3_/Bi_2_S_3_ 10cyc	7.12	13.83	18.59	2.09	43.38	-	14.99

**Table 2:** Photoelectrochemical parameters of the photoelectrodes from Zview fitting of EIS.

Electrode	R_ct_ (W cm^2^)	C_CPEct_ (W^−1^s^n^cm^−2^) × 10^−4^	R_b_ (W cm^2^)	C_CPEb_ (W^−1^s^n^cm^−2^) ×10^−4^	R_f_ (W cm^2^)	C_CPEf_ (W^−1^s^n^cm^−2^) ×10^−4^	R_p_ (W cm^2^)
WO_3_	8007.0	34.683	-	-	-	-	8007.0
WO_3_/α-Fe_2_O_3_	283.3	20.390	-	-	576.0	4.224	859.3
WO_3_/α-Fe_2_O_3_/Bi_2_S_3_ 4cyc	2.2	2.174	218.8	7.563	539.8	17.411	760.8
WO_3_/α-Fe_2_O_3_/Bi_2_S_3_ 6cyc	2.8	49.322	142.0	40.315	105.0	11.220	249.8
WO_3_/α-Fe_2_O_3_/Bi_2_S_3_ 8cyc	0.5	50.598	1.6	1.049	98.2	9.679	597.9
WO_3_/α-Fe_2_O_3_/Bi_2_S_3_ 10cyc	1.0	35.489	62.4	32.373	262.5	4.044	325.9

**Table 3:** *V_fb_, N_d_*, and W values of the photoelectrodes.

Photoelectrodes	V_fb_ (V_RHE_)	N_d_ (cm^−3^) × 10^20^	W (nm)
WO_3_	0.533	3.31	19.4
WO_3_/α-Fe_2_O_3_	1.389	63.70	15.3
WO_3_/α-Fe_2_O_3_/Bi_2_S_3_ 4cyc	1.357	16.30	29.1
WO_3_/α-Fe_2_O_3_/Bi_2_S_3_ 6cyc	1.339	12.10	33.9
WO_3_/α-Fe_2_O_3_/Bi_2_S_3_ 8cyc	1.334	8.29	41.4
WO_3_/α-Fe_2_O_3_/Bi_2_S_3_ 10cyc	1.054	7.69	33.3

**Table 4:** State-of-the-art ternary photoanodes in OER process.

Electrode	Electrolyte	Illumination power	Photocurrent density (mA cm^−2^)	Ref
WO_3_/α-Fe_2_O_3_/Bi_2_S_3_	0.1M Na_2_S and 0.1M Na_2_SO_3_	100 mW cm^−2^	5.777 mA cm^−2^ measured at 1.0 V vs. RHE	This work
WO_3_/α-Fe_2_O_3_/NiFe-LDH	1.0 M NaOH	100 mW cm^−2^	3.0 mA cm^−2^ measured at −1.8 V vs. RHE	[50]
WO_3_/BiVO_4_/NiFeCr-LDH	0.1 M PBS	100 mW cm^−2^	4.9 mA cm^−2^ measured at −1.8 V vs. RHE	[51]
α-Fe_2_O_3_/WS_2_/WO_x_	0.1 M NaOH	100 mW cm^−2^	2.1 mA cm^−2^ measured at −0.47 V vs. RHE	[28]
WO_3_/CuWO_4_/CuO	0.5 M Na_2_SO_4_	100 mW cm^−2^	2.24 mA cm^−2^ measured at −1.23 V vs. RHE	[52]
